# Children’s Exposure to Environmental Contaminants: An Editorial Reflection of Articles in the IJERPH Special Issue Entitled, “Children’s Exposure to Environmental Contaminants”

**DOI:** 10.3390/ijerph13111117

**Published:** 2016-11-09

**Authors:** Alesia Ferguson, Helena Solo-Gabriele

**Affiliations:** 1 Environmental and Occupational Health, Fay W. Boozman College of Public Health, University of Arkansas for Medical Sciences, 4301 West Markham, Slot 820, Little Rock, AR 72205, USA; Aferguson@uams.edu; 2 Department of Civil, Architectural, and Environmental Engineering, College of Engineering, University of Miami, Florida, 1251 Memorial Drive, Coral Gables, FL 33146, USA

**Keywords:** children’s exposures, children’s health, risk assessment methods, environmental influences

## Abstract

Children are at increased vulnerability to many environmental contaminants compared to adults due to their unique behavior patterns, increased contaminant intake per body weight, and developing biological systems. Depending upon their age, young children may crawl on the floor and may practice increased hand to mouth activity that may increase their dose-intake of specific contaminants that accumulate in dust and other matrices. Children are also smaller in size than adults, resulting in a greater body burden for a given contaminant dose. Because children undergo rapid transitions through particular developmental stages they are also especially vulnerable during certain growth-related time windows. A Special Issue was organized focused on the latest findings in the field of children’s environmental exposure for these reasons. This editorial introduces articles in this Special Issue and emphasizes their main findings in advancing the field. From the many articles submitted to this Special Issue from around the world, 23 were accepted and published. They focus on a variety of research areas such as children’s activity patterns, improved risk assessment methods to estimate exposures, and exposures in various contexts and to various contaminants. The future health of a nation relies on protecting the children from adverse exposures and understanding the etiology of childhood diseases. The field of children’s environmental exposures must consider improved and comprehensive research methods aimed at introducing mitigation strategies locally, nationally, and globally. We are happy to introduce a Special Issue focused on children’s environmental exposure and children’s health and hope that it contributes towards improved health of children.

## 1. Introduction

The federal government recognized infants and children as a special group to consider for their cumulative and aggregate exposures to pesticides under the United States Food Quality Protection Act in 1996 [1]. The Act recognized that children had unique exposure behaviors and that lower doses of any contaminant could have greater adverse effects on their growth, development, and survival. In essence, children were not to be treated as mini adults where a simple relative factor was used to adjust for exposure and dose metrics. This was followed in 1997 by a more general executive order, “The Protection of Children from Environmental Health Risks” [2]. Since the enactment of these laws, there have been increased activities in the United States and around the world to study children’s environmental exposures and address mitigation strategies to protect children. The biological, chemical, and physical contaminants of most concern include those that children likely encounter in the indoor and outdoor environment. Increased consideration should also be given to those contaminants found in high concentrations in a child’s environment and particularly those that are highly toxic. Schools and homes are indoor environments where young children spend the most time, but even those environments are directly affected by geographic region, cultural practices, and varying economic and political influences (e.g., civil unrest, overcrowding, and poverty). Therefore, for every geographical region or country, the list of contaminants and the potential exposures may vary in order of significance and possible impact on children’s health.

As a result of the continued need to focus specifically on children’s health, a Special Issue entitled, “Children’s Exposure to Environmental Contaminants” was initiated to consolidate new knowledge focused specifically on children and the environment. The issue aimed to showcase and summarize new information about child behaviors that are useful for quantifying exposures of children to environmental contaminants, children’s susceptibility to contaminants at various growth stages, and contaminant distributions in areas that present exposure risks to children. Innovative risk assessments focused on children’s susceptibility to environmental contaminants were also welcomed. As a result, a total of 23 papers were reviewed and accepted for publication in this Special Issue. The articles have been grouped for this editorial according to the following themes where some overlap in categories exists: exposures to ionizing radiation, exposures to metals, exposures to pesticides and other organic compounds, exposures linked to respiratory irritants, and techniques and frameworks to better measure and quantify children’s exposures.

## 2. Articles in This Special Issue

We are excited to present a Special Issue focused on children’s exposures to environmental contaminants that features 18 full length research articles, 1 case study and 4 communications. Special Issue author names are bolded in this editorial to distinguish them from other articles referenced. These papers collectively explore new environments, techniques, study designs, and populations. With respect to the specific contaminants, three of the articles are focused on children’s exposure to radiation. Two of those articles address exposure to radon gas where radon exposure entails a mechanistic action of daughter radon elements attaching to particles in the air which are then inhaled [3]. Branco et al., considers the structural, soil, and human use aspects of school and nursery buildings that may influence measured radon gas infiltration and therefore indoor radon concentrations and potential exposure for children in two districts of Portugal [4]. The authors find some exceedance of internal recommendations of indoor radon levels and emphasize that radon accumulation is a function of building occupancy, ventilation and movement, classroom floor level and the age of the building [4]. Peckham et al., considers a large retrospective epidemiological study to evaluate associations between indoor residential radon levels in the state of Texas and childhood lymphoma, finding low to no evidence in keeping with previous findings, as the authors indicate [5]. Only a marginal non-significant increase in a specific type of lymphoma (i.e., non-Hodgins) was observed in areas with the higher radon concentrations. The third article on radiation is a review article by Kutanzi et al. This article focuses on pediatric exposures to ionizing accidental, technological and diagnostic radiation and the potential for carcinogenic outcomes given existing radiosensitivities [6]. The authors emphasize that the role of genetic sensitivities during and following radiation exposures needs to be further explored [6,7].

Children’s exposure to metals and their potential to influence adverse health continues to interest researchers globally. We published four articles that addressed exposures to metals in this Special Issue. Yeter et al., explores various ethnic groups for the association between low-dose mercury and cadmium exposure due to consumption of seafood and pediatric Kawasaki disease in US children, finding a need to further research potential links [8]. Kawasaki disease, the second leading cause of heart attacks in children under five years of age, primarily affects ethnic populations (i.e., Asian and Blacks), and its etiology continues to perplex researchers in the field [9]. Shibata et al., conducted a risk assessment using U.S. Food and Drug Administration (FDA)-identified arsenic levels in associated children’s dietary products (i.e., rice cereal) along with recommended daily intake rates for these products, determining some concern for chronic exposures to children in the U.S. [10]. In a study of 68 children in Arizona, Beamer et al., found associations between urinary levels of a secretory protein (i.e., CC16, a potential clinical biomarker for airway damage due to exposure to arsenic) and arsenic concentration in soil, water, house-dust and dust loadings [11]. In this study an association with soil arsenic remained when other environmental and behavioral factors were controlled for in the model. The effect of low level exposures of lead on learning disabilities have been routinely studied [12]; however, Blackowicz et al. take a very specific look at this relationship in Hispanic subgroups in the Chicago area [13]. Authors suggest that 7% of reading and 13% of math failure may be attributable to blood lead levels (BLLs) of 5–9 µg/dL, with an influence greater for black Puerto Rican children over white Puerto Rican children [13].

There is a multitude of ever-changing pesticides and other organic compounds used in our environment and it is practical to continue to assess their impact on children’s health. The research on pesticide exposures in the home and their impact on children’s health experienced a shift in the class of pesticides under scrutiny based on registration usage changes in various countries. In the United States there was greater concern in previous years for organophosphate (OP) use in the home and its impact on children’s health (e.g., neurological outcomes, cancers) before the phase out period between 2001 and 2004 [14]. Although OP residues are persistent indoors, there is now additional focus on the newer class of pesticides called pyrethroids and the resulting exposures and potential health outcomes for children (e.g., allergies) [15]. 

Given the growing number of children with Autism Spectrum Disorders (ASD) over the last 20 years, Domingues et al. comparatively measure the level of the pyrethroid metabolite 3-PBA in urine, and metals and microelements in hair for ASD and non-ASD children in Italy aged 5 to 12 years [16]. There were some marginally significant increases for some metals and 3-PBA for ASD; however further studies are warranted in order to make any definitive association. Autism is a neurological disease for which environmental exposures are believed to play a role, and for which comorbidities, such as immune system dysfunction and other psychiatric problems, are common [17]. There has long been an understanding among researchers that age of parents and heredity are strong predictors for genetically prone individuals. Polychlorinated biphenyls (PCBs), polybrominated diphenyl ethers (PBDEs), heavy metals, pesticides, small particulate pollution, viral and bacterial exposure during critical windows of pregnancy, and immune dysfunction in the mother are also suspected to contribute to autism outcomes [17]. The only case study in this Special Issue used measured pesticide metabolites in the child’s urine to link facial paresthesia in a 13-month old to recent well documented indoor use of pyrethroids in the home to treat ants. By all indications pesticide use occurred according to label recommendations indicating increased care is warranted by the public in the use of pesticides around the home as recommended by Perkins et al. [18].

There continues to be concern for exposures for pregnant women and children that live on or close to farms, due to drift and track-in of multiple classes of pesticides; studies on these pesticides have shown mixed results for various cancers and reproductive and neurological outcomes [19,20,21]. With the heavy use of pesticide on agricultural farms in China, Silver et al. looked for the presence of multiple classes of pesticides in the umbilical cords of 336 infants, finding evidence for individual exposures to multiple pesticides and increased levels for those born in the summer months [22]. Rahbar et al., also detected 1 of 17 organochlorine (OC) pesticides (i.e., 4,4′-DDE hexane) in the umbilical cord blood serum in a study of 67 Jamaican newborns, where 37% of the samples were above the limit of detection (LOD) [23]. In this study, limited concentrations of PCB congeners (i.e., PCB-153, PCB-180, PCB 206) were also detected. Although the study is based on a limited dataset, new information on pesticides and other organics is provided for an understudied group of Caribbean children where an investment in a larger study is warranted.

Housedust contaminant loading and content—affected by proximity to industry, smoking activities, infiltration of outdoor soils, products used in homes, and the age of the home—are considered dominant sources of exposure to many contaminants, including a variety of organics [24,25]. PBDEs are also commonly recognized for their use as flame retardants in household and consumer products. Shin et al. explore the association of PBDE congener concentrations in housedust with PBDE congener levels in tissues of mother-baby pairs, finding some positive associations [26]. The authors provide a radar chart to view the distributional comparison among congeners. Their study highlights housedust as an exposure source of chemicals for pregnant women and for children, and suggests the need for strategies to mitigate this exposure pathway for organics and other compounds.

The field of children’s environmental exposures with respect to respiratory irritants and their sources continues to develop with some informative articles in this series. Data from the United States National Children’s Study, looking at 28 VOC metabolites in the urine of 488 third trimester pregnant women, are compared to possible sources of VOC exposures [27]. Cigarette smoke, paint use, and incense from indoor exposures have varying levels of significance in the analysis as indicated by Boyle et al. In another Special Issue paper, Mohammed et al. addressed the potential link between exposure to environmental nitric acid and respiratory syncytial virus (RSV) admission data. Nitric acid is considered a volatile inorganic acid. No association was found in this study. However, this lack of association may be attributable to the low number of events in the studied time frame and the authors suggest a more comprehensive study [28]. McInnes et al. showed that bronchial airway epithelial cells removed from children exhibited increased release of pro-inflammatory interleukins (IL-6 and 8) following exposures to house mite allergens, while interestingly showing lower levels of IL-6 for higher versus lower levels of side-stream cigarette smoke [29]. The rising rates of children’s asthma and allergies also concern public health officials and researchers in the United States and in other countries, as we should continue to address agents and sources of exposures [30,31]. 

Some authors explored specific environments of exposure for children in this Special Issue (i.e., beaches, parks, and farms). Although inhalation exposure is the primary concern in outdoor environments, soils in playgrounds, parks and beaches may be contaminated with pollutants like the metals arsenic and lead [32], putting children at risk for exposure also through the dermal and non-dietary ingestion exposure routes. Our Special Issue paper on beach exposures by Black et al., highlighted the many contaminants (e.g., metals, aromatic hydrocarbons) that can be found following environmental accidents such as the Deep Horizon spill. Black et al., conducted a deterministic risk assessment to these oil spill chemicals for children, identifying low risk based on the Environmental Protection Agency (EPA) monitoring data during and after the Deepwater Horizon disaster and other measurements five years later [33]. Although, based on the deterministic risk assessment, the acute, sub-chronic and cancer risks were deemed low for children at the time, the authors of the paper emphasized the lack of toxicological data for dispersants and oil spill degradation products and the lack of human activity data needed to estimate exposures of children during recreational beach use [33].

Areas where children play can be a cause for concern also for infectious disease transmission. In this Special Issue, particular attention is given to ornamental waters (i.e., water fountains) in parks in Portugal by Nascimento et al. [34]. The authors show a dynamic microbiota present with potential for increased antibiotic resistance, where antibiotic resistance is associated with the presence of biofilms. Other studies in the field have also shown the accumulation of infectious disease agents in sands used by children for play activities. These include sands in intertidal zones at beaches [35,36] and also in sandboxes [37]. Within the intertidal zone, the area just above the high tide line is where the highest bacteria levels have been observed, and is the area where children tend to play. These bacteria levels correlate with protozoa and helminth levels suggesting potential exposures to pathogens in this area [37]. A follow-up study on children’s activity patterns in these environments and an assessment of health risks is encouraged by these authors and others in the field.

A combination of factors has played a role in the increasing rates of diseases like asthma and autism, including increased rates of diagnosis [17]. Research is ongoing, however, on etiology and the changing environment that may be influencing outcomes and rates. There are some common findings among studies for causal and related factors, such as positive association of a child’s asthma outcome with atopy, parental atopy and asthma, indoor and outdoor pollution, and a negative association with living in a rural area [38,39,40,41]. Based also on the weight of collected evidence, the Institute of Medicine has declared that house dust mite (HDM) allergens, cockroach allergens, cigarette smoke, and the RSV are causal in the development of asthma [42]. Numerous environmental chemical and biological agents have also been declared as triggers, including dampness, mold, and household VOCs [43]. Some of our Special Issue papers above explored air pollution sources or agents and their potential influence on respiratory diseases, including asthma (e.g., [29]). The asthma-rural hypothesis was further supported in this Special Issue through Timm et al. [44]. Using a population-based study in Europe, Timm et al., observed an urban-rural protective gradient, where early life exposures on farms were advantageous in the prevention of asthma development.

A complete human risk assessment considers the source, fate and transport, exposure, dose and uptake, and the associated health risks of the agent, be it chemical, biological, or physical [45]. Direct approaches to determine exposure include personal monitors (e.g., patches, personal air monitors), while indirect approaches consider use of algorithms or models to estimate exposures. Modeling approaches can be data-intensive, but they allow for estimates of exposures and doses over time, and even the ability to conduct sensitivity analysis to determine critical and risk-modifying parameters. Exposure, dose, and health risk assessments for children approach greater accuracy when children or parameters directly related to children are considered and incorporated [46]. In this Special Issue, Ginsberg et al., describe a four-step methodology for exposure assessment. This methodology synthesizes available information to identify key sources of early life exposure to contaminants. The four step process focuses on prioritization of life stages and exposure pathways, estimation of pathway-specific exposures, comparison of aggregate exposure by pathways with bio-monitoring-based approaches, and source apportionment [47].

Human activity patterns are crucial determinants of exposures, where the level of detail (i.e., micro, mesa, and macro) in the activity patterns are determined by the toxicological endpoint, route of exposure (i.e., inhalation, ingestion, dermal), time integral, and the indirect or direct modeling approach of interest [48,49]. The activities of children are highly variable and are influenced by the larger environment (i.e., country and weather) and also the immediate environment (i.e., home and culture). For example, in developing countries, infants are carried on their mothers’ backs while cooking over wood stoves, directly exposing them to harmful VOCs [50]. Kwong et al. present an informative paper on the hand- and object-to-mouth activity patterns of 148 children in Bangladesh, where a child’s environmental surroundings and familial cultural practices may influence exposure activities and therefore biological and chemical health risks [51]. The authors find significant higher hand- and object-mouthing frequencies for children in Bangladesh in comparison to children in the U.S., concluding that exposure models from U.S. and other high-income countries might not accurately estimate children’s exposure to environmental contaminants in other countries [51]. A better understanding of how children behave in the indoor and outdoor environment improves the understanding and the estimate of health risks [15].

There are a number of published approaches to children’s chemical health risk assessments. In 2000, Armstrong and colleagues developed a formalized tiered approach for addressing the highest potential chemical risks for children and later presented methods and data for that tiered approach [52]. Georgopoulos et al. have also developed a tiered framework for ranking chemical exposures based on extant environment, demographic and socio economic data [53]. Other researchers have presented more detailed algorithms and consideration of relevant factors for addressing specific exposure scenarios (e.g., [54,55]). In this Special Issue, Smith et al. developed a toxicological framework for the Washington Children’s Safe Products Act (CSPA) to score and rank the importance of 66 chemicals based on toxicological endpoints, chemical properties, and exposure potential, finding formaldehyde, dibutyle phthalate, and styrene to be the highest priority chemicals [56]. Susceptibility and life stages for children are considered in their assessment. Children’s products potentially contain numerous chemicals that might put them at risk for exposures as evidenced by the CSPA and the importance of this prioritizing tool is evident in this article. Other prioritization tools are presented in the article for comparison (e.g., EPA ExposCast and the Toxicological Prioritization Index (Toxpi)) [56].

In any modeling approach, representative environmental concentrations are imperative for accurate estimates of exposure and resulting health risk outcomes. Continuous measures of environmental concentrations where the child is located are ideal [50]. However, this is not always possible or feasible, especially in retrospective studies. Various strategies to extrapolate chemical, biological, or physical concentrations are employed. For air pollution, proxy measurements are utilized along with dispersion and land use modeling to estimate the concentration in a child’s more immediate environment [50]. The ability to better measure children’s exposures to particulate matter is presented by Shah et al., in this Special Issue by the use of a field robotic sampler able to mimic children’s activity patterns in the home [57]. By using the sampler, the authors also show a potential link of exposures to particulate matter in the home and eczema in a group of 128 children. The last paper in the Special Issue by Kumar uses a study of birth weight and particulate matter in Chicago to address the potential exposure uncertainty that results from dissimilarities in spatiotemporal resolution in environmental and health data [58]. The authors provide strategies to minimize uncertainty by selection of applicable distance and time-intervals.

## 3. Conclusions

Given the large response in papers submitted and ultimately published in this Special Issue, a considerable amount of research is being conducted in the area of children and environmental contaminants. These studies collectively advance the field but many gaps still exist in the context of the contributions of the papers in the Special Issue and the literature as a whole. The field of children’s exposure is complex and in a state of flux with changing environments, changing behaviors, and changes in the manufacture and use of consumer, agricultural, and building products. Determining a cause and effect relationship for any health outcome relies on a systematic and comprehensive evaluation of all exposure and risk factors. In reviewing studies in the field, one must be careful to discern differences in study intent, design, and findings. Caution needs to be taken in understanding associations versus causal factors, given the weight of evidence in the field. The onus of responsibility is on the researchers to represent limitations and bias within any study, along with potential variability across space and time and any potential uncertainty in measurements and modelling computations. Underlying biological mechanisms and confounding factors (e.g., gene variants, multiple exposures, poverty, poor nutrition, predisposing and enabling factors natural, built and social aspects) can provide the audience with a broader understanding of the complexity of certain health outcomes [53,59]. Certainly, attention must be paid to how we represent data and communicate the risks to the public [60]. This Special Issue contributes to understanding some of the nuances associated with children environmental exposures in the context of radiation, metals, pesticides and other organic compounds, respiratory irritants, specific areas of exposure, and identification of frameworks for assessing exposure and risk. Additional research is needed, however, to continue to address the knowledge gaps in this important area of children’s health. 
